# Self-Compassion and Psychological Health of Parents: A Meta-Analysis Focused on Some Neurodevelopmental Disorders

**DOI:** 10.1007/s10803-025-06841-9

**Published:** 2025-04-21

**Authors:** Cansu Ozturk, H. Senay Guzel

**Affiliations:** 1 Faculty of Health Sciences, Department of Speech and Language Therapy, Yuksek Ihtisas University, Oğuzlar District, 1375th Street, No:8, 06520 Cankaya, Ankara, Türkiye; 2 Faculty of Humanities and Social Sciences, Department of Psychology, Ankara Yildirim Beyazit University, Dumlupinar District, 06760 Cubuk, Ankara, Türkiye

**Keywords:** Psychological distress, Parental well-being, Parenting stress, Self-compassion, Neurodevelopmental disorders, Autism

## Abstract

Parents of children with neurodevelopmental disorders (NDDs) may face various psychological problems and experience parenting stress because of the nature of these disorders. Parents’ psychological distress and high levels of parenting stress have a negative impact on their well-being. In addition, recent studies on the buffering effect of self-compassion related with psychological difficulties have also attracted attention. At this point, this study aims to carry out a meta-analytic review of studies examining the association between self-compassion and psychological distress, parenting stress, and well-being. Web of Science (WOS), Scopus, and EBSCOHost (APA PsycArticles, MEDLINE, TR Index, ERIC) electronic databases were searched in November 2023. Studies were included if they were quantitative and included parents of children with NDDs as the study population. As a result, 131 studies were obtained. After the duplicate studies were removed and evaluated according to the inclusion criteria, *n* = 15 were included. The random effects model was used to obtain the pooled effect sizes. The results showed that there was a large, negative, and significant relationship between self-compassion and parental depression score and parenting stress; a moderate, negative, and significant relationship between self-compassion and parental anxiety score; and a large, positive, and significant relationship between self-compassion and parental well-being. According to these findings, it is important to observe and measure the level of self-compassion for the well-being of parents of children with neurodevelopmental disorders. Moreover, increasing self-compassion in parents of children with neurodevelopmental disorders may be a protective factor for the psychological health of these parents.

**Supplementary Information:**

The online version contains supplementary material available at https://doi.org/10.1007/s10803-025-06841-9.

According to the Diagnostic and Statistical Manual of Mental Disorders, 5th Edition, Text Revision (DSM-5-TR), neurodevelopmental disorders (NDDs) include intellectual disability (ID), communication disorders (CD), autism spectrum disorder (ASD), attention-deficit/hyperactivity disorder (ADHD), specific learning disorder (SLD), and motor disorders (MD) (APA, 2022). These disorders emerge with distinct symptoms in early childhood, endure throughout life, and have overlapping characteristics; thereby, they are categorized as neurodevelopmental disorders (Francés et al., 2022; Martelli et al., 2025; Thapar et al., 2017). However, they also display significant differences (Martelli et al., 2025). As it was stated, ASD, ADHD, and ID share a common set of symptoms, primarily characterized by impairments in communication, social interactions, attention, and executive functioning when they are compared with other NDDs (Emmanuel et al., 2022; Pedersen et al., 2017; Thapar et al., 2017). In the study by Martelli et al. (2025), children with ASD exhibited lower scores in language and communication skills, social-emotional development, eye-hand coordination, and learning abilities compared to those with language disorders. However, they demonstrated higher levels of affective problems and attention-deficit/hyperactivity symptoms. In contrast, children with language disorders performed better in daily living skills, motor skills, social skills, and communication abilities. Moreover, when compared to ASD and Tourette Syndrome, despite similarities such as hand mannerisms, social isolation, and poor imaginative play, ASD appears to differ in aspects such as special interests, self-harming behaviors, ritualistic actions, and lack of social imitation (Eapen et al., 2019). In addition, the co-occurrence of ASD and ID seems to accelerate the extent of behavioral and emotional difficulties in children (Totsika et al., 2011). While neurodevelopmental disorders share common features, they also exhibit distinct differences in symptomatology, developmental impact, and functional outcomes.

Considering the diverse challenges and heterogeneity associated with NDDs, it is essential to adopt a comprehensive perspective that addresses the affected children’s experiences and acknowledges the broader impact on their families (Norton & Drew, 1994; Rosenbaum & Gorter, 2012). In line with this perspective, Rosenbaum and Novak-Pavlic (2021) critique the prevailing tendency among professionals to primarily focus on children with NDDs, often overlooking the perspectives and well-being of their parents.

A review of studies on parents of children with NDDs indicates elevated levels of parenting stress (Craig et al., 2016), social stigma (Kinnear et al., 2016), depression and anxiety symptoms (Chan & Leung, 2021; Fırat, 2016; Ingersoll et al., 2011), marital conflicts (Hartley et al., 2018), and financial difficulties (Parish et al., 2015) within these families. Moreover, parents of children with NDDs may perceive their parenting self-efficacy as lower (Pointer, 2015) and experience reduced levels of well-being (Lee, 2013). However, parental experiences with NDDs may vary depending on the specific type and severity of the disorder (Dovgan & Mazurek, 2018; Emmanuel et al., 2022). Dovgan and Mazurek (2018) suggest that raising a child with ADHD, ASD, or ID is associated with varying types and intensities of perceived burden. Notably, their findings indicate that parents of children with ASD or ID experience higher levels of financial, psychological, and logistical stress compared to those parenting children with ADHD.

Despite the challenging nature of NDDs, it is also possible to mention the protective role of some parent-related variables, such as self-compassion. The concept of self-compassion is rooted in Buddhist teachings and is defined by Neff (2003a) as a healthy alternative to approaching oneself. Self-compassion is conceptualized with three critical elements: “self-kindness,” “common humanity,” and “mindfulness.” The first element, self-kindness, is about approaching oneself compassionately and sympathetically instead of judging oneself excessively when experiencing pain or failure (Neff, 2003a). In other words, self-kindness can be conceptualized as an approach that emphasizes self-care, self-nurturance, and the acceptance of one’s imperfections (Coyne et al., 2021). Common humanity refers to recognizing that an individual’s experiences are shared by others rather than perceiving them as unique or isolated occurrences (Neff,)2003a). In this context, self-compassion comes from understanding that our own experience is a condition unique to all of humanity (Coyne et al., 2021). Mindfulness involves maintaining a balanced awareness of unpleasant thoughts and emotions rather than over-identifying with them (Neff, 2003a). However, it also consists in recognizing and being present with suffering—both our own and that of others—rather than ignoring, suppressing, or avoiding it (Coyne et al., 2021). In conceptualizing self-compassion, Neff (2003a) states that self-compassion does not mean being passive or ignoring one’s shortcomings. Instead, according to her, self-compassion leads to growth and development by enabling the individual to gain self-awareness.

Furthermore, self-compassion inspires people to give up harmful behavioral patterns and motivates them to adopt more positive attitudes toward their well-being (Neff, 2003a). Research suggests that self-compassion is significantly associated with exhibiting kindness and acceptance toward oneself, particularly in challenging circumstances. This construct of self-compassion functions as a protective mechanism, mitigating the impact of anxiety and fostering healthier self-perceptions (Leary et al., 2007; Neff et al., 2007).

Much recent research has explored the relationship between self-compassion and many facets of parenting (Jefferson et al., 2020). According to studies, self-compassion is negatively related to parental shame and guilt feelings (Sirois et al., 2019), parental burnout (Kroshus et al., 2023), stress (Mancini et al., 2023), depression (Felder et al., 2016), and anxiety (Beer et al., 2013). Moreover, parents who practice self-compassion tend to adopt a more beneficial parenting style (Gouveia et al., 2016), engage in mindful parenting (Beer et al., 2013; Nguyen et al., 2020), and demonstrate greater self-efficacy in their parenting practices (Mancini et al., 2023). The relationships between self-compassion and psychological distress, parenting stress, and well-being will be further discussed in the following part in connection to the subject of this meta-analysis.

## Self-Compassion and Psychological Distress

Psychological distress involves undesirable emotions (Feng et al., 2020) and is often conceptualized in terms of depression, anxiety, and stress symptoms (Chan et al., 2020a, b; Robinson et al., 2018; Satici et al., 2021). Research indicates that parents of children with NDDs experience significantly higher levels of psychological distress compared to parents of typically developing children (Lach et al., 2009; Maridal et al., 2021; Stanford et al., 2022). Moreover, there can be differences depending on factors such as the type of NDDs, symptom severity, whether the child has feeding problems, and whether the parent is receiving professional help (Maridal et al., 2021; Nagase et al., 2024). According to Lach et al.‘s (2009) study, having a child with both a neurodevelopmental disorder and externalizing problems is associated with higher levels of depression in mothers. It was also shown that mothers of children with ASD had higher depression scores compared to those with ADHD and ID (Nagase et al., 2024).

Pauley and McPherson (2010) suggest that although individuals experiencing symptoms of depression and anxiety tend to show compassion toward others, they often struggle to extend the same compassion to themselves. Numerous studies have shown a negative relationship between self-compassion and symptoms of depression, anxiety, and stress; in other words, lower levels of self-compassion are associated with higher levels of psychological distress (De Souza et al., 2020; Deniz & Sümer, 2010; Muris et al., 2016; Özyeşil & Akbağ, 2013; Soysa & Wilcomb, 2015). Given this established link between self-compassion and psychological distress, research has also explored its significance in the context of parenting. According to the literature, parents’ level of self-compassion is also related to anxiety and depressive symptoms (Beer et al., 2013; Chan et al., 2020a, b; Ivins-Lukse & Lee, 2021; Psychogiou et al., 2016). Psychogiou et al.‘s (2016) study found that parents with higher self-compassion show lower depressive symptoms and exhibit a more positive parenting approach. Another study reported that mindful parenting, which includes non-judgmental acceptance and emotional awareness, is negatively related to parents’ depressive symptoms (Beer et al., 2013). Supporting parents of children with ADHD in a mindful parenting group emphasizing self-compassion has also been shown to have a positive effect on parents’ anxiety and depression symptoms (Liu et al., 2021). In addition, Robinson et al. (2018) found a significant negative correlation between self-compassion levels and depression and stress levels of parents of individuals with intellectual disability and developmental disorder (IDD). As a prominent finding in the same study, it was observed that the self-compassion levels of these parents were not associated with their children having an additional ASD diagnosis or another psychiatric diagnosis.

### Self-Compassion and Parenting Stress

Parenting can be challenging for most parents, as each individual has limited resources—mainly when a child exhibits developmental patterns or characteristics that deviate from the norm (Abidin, 1990a). Parenting stress refers to negative feelings toward oneself or the child that arise directly from the demanding nature of parenting (Deater-Deckard, 1998). In addition to the demanding nature of parenting, the psychological state of the parent, the level of the parent-child relationship, and the child’s psychosocial adaptation are other factors related to parenting stress (Deater-Deckard, 1998). Craig et al. (2016) state that recent advances in the study of parenting stress brought back interest in the reciprocal relationship between parenting stress and NDDs. It can be argued that having a child with NDDs - for example, ASD- can involve additional parenting stress in terms of social problems, difficulties in behavior management, problems with self-care skills, and concerns about the child’s physical health and future (Silva & Schalock, 2012). When the research is taken into account, it becomes clear that certain studies have found a relationship between high levels of parenting stress and having a child with NDDs (Ashworth et al., 2019; Baker & McCal, 1995; Schieve et al., 2007; Theule et al., 2013). However, it is essential to keep in mind that the experience of parenting stress may vary depending on the subtype of NDDs (Craig et al., 2016). In their study, Baker and McCal (1995) found that children with ADHD exhibit more externalizing problems than those with SLD and that mothers of children with ADHD experience higher levels of parenting stress compared to mothers of children with SLD. Similarly, Craig et al. (2016) demonstrated that parents of children with ASD and ADHD experience the highest levels of parenting stress when compared to parents of children with SLD and those without NDDs. Furthermore, in a study examining parenting stress across different NDD subtypes, Stewart et al. (2015) reported that parenting stress was more strongly associated with the comorbidity of ADHD and obsessive-compulsive disorder in children with Tourette syndrome rather than the severity of the child’s tics. Parenting stress has also been found to be associated with the presence of multiple co-occurring neurodevelopmental disorders. While mothers of adolescents with ASD and those of adolescents with ADHD did not exhibit significant differences in parenting stress levels, mothers of adolescents with both ASD and ADHD experienced significantly higher levels of parenting stress (Schiltz et al., 2022).

While parenting stress may be particularly noticeable among parents of children with some subtypes of NDDs, it is essential to remember that parenting stress can vary depending on how parents explain and address the problem (Craig et al., 2016). According to research, parenting stress is inversely correlated with self-compassion, which can be considered a coping strategy (Garcia et al., 2022; Gouveia et al., 2016; Stenz et al., 2023). For example, Stenz et al. (2023) found a negative correlation between parenting stress and self-compassion when the child has psychological disorders. Gouveia et al.‘s (2016) study demonstrated that higher levels of mindful parenting, associated with lower levels of parenting stress, are related to proneness to mindfulness and self-compassion. Furthermore, Riany and Ihsana (2021) found that parents of children with ASD and ADHD experienced lower levels of parenting stress if they were self-compassionate and perceived higher levels of social support. Torbet et al. (2019) found that social support and symptom severity predicted parenting stress. Still, when self-compassion, public, and affiliated stigma variables were added to the next steps in the hierarchical regression analysis, social support lost its predictive role, and symptom severity was the largest predictor of the unique variance in parenting stress, followed by self-compassion, affiliated stigma, and public stigma. While the findings of Torbet et al. (2019) highlight the significant predictive role of self-compassion in parenting stress, they also underscore the importance of considering ASD-related symptoms and social factors in understanding the parenting stress experienced by caregivers.

### Self-Compassion and Parental Well-Being

Psychological well-being is defined as an individual’s experience of ‘feeling good’ and functioning effectively on a psychological level. In this context, ‘feeling good’ encompasses happiness, life satisfaction, a sense of care, commitment, trust, and love. Effective functioning is associated with developing one’s potential, having control over one’s life, having a sense of purpose, and building satisfying relationships with others (Huppert, 2009). However, parenting a child with NDDs can pose significant challenges to these aspects of well-being (Moen et al., 2016). For example, Lai et al. (2015) found that parents of children with ASD reported lower levels of well-being compared to parents of typically developing children. Furthermore, parental emotional well-being is strongly impacted by factors such as the child’s age and the presence of comorbid NDDs, with younger children and ASD/ADHD comorbidity presenting greater challenges (El Hajri, 2024; Romaniuk et al., 2024). Beyond the child’s diagnosis, the parent’s predisposition to or diagnosis of an NDD can further diminish their well-being. In their study, Moen et al. (2016) found that parents with ADHD reported significantly lower levels of well-being compared to those without ADHD.

Research on self-compassion and well-being has shown a positive correlation between these variables, suggesting that higher levels of self-compassion in parents are associated with greater well-being (Nemati et al., 2023; Tekinarslan & Tok, 2023; Torbet et al., 2019). In their study with mothers of children with intellectual disabilities, Tekinarslan and Tok (2023) found that self-compassion significantly predicted mothers’ well-being directly and through psychological resilience. In the same study, social support was found to predict well-being only through the mediating role of psychological resilience. In another study, Nemati et al. (2023) examined the relationships between self-compassion, psychological resilience, and well-being in mothers of children with ASD. They found that self-compassion was the most important predictor of well-being. Torbet et al. (2019) found that social support was the largest predictor of the unique variance in well-being, followed by self-compassion and symptom severity. However, in addition to individual and psychological factors, social dynamics also play an important role in shaping parental well-being. Emmanuel et al. (2022) found that parents, particularly those from minority backgrounds or those who hold differing views from their close family members regarding their child’s diagnosis and treatment, often experience social distancing and isolation. This social exclusion may, in turn, erode their self-compassion and overall well-being, highlighting the need for a more comprehensive approach that considers both psychological and social determinants.

### Current Meta-Analytic Review

As stated above, having a child diagnosed with NDDs is related to some negative consequences for parents (Chan & Leung, 2021; Craig et al., 2016; Hartley et al., 2018; Ingersoll et al., 2011; Kinnear et al., 2016). However, individual differences in how parents approach the situation, such as showing self-compassion, may buffer against these adverse effects (Gouveia et al., 2016; Liu et al., 2021; Stenz et al., 2023; Torbet et al., 2019; Zessin et al., 2015). Screening of the literature showed that there are studies investigating the association between parental self-compassion and psychological distress, parenting stress, and well-being (Chan et al., 2020a, b; Ivins-Lukse & Lee, 2021; Neff & Faso, 2015; Pyszkowska et al., 2021; Tekinarslan & Tok, 2023; Torbet et al., 2019). Given that individual studies often rely on relatively small samples, one of the primary purposes of meta-analyses is to enhance the power and precision of parametric estimations by integrating these findings into a more comprehensive and meaningful large-scale analysis (Kürü, 2021). When individual studies in the literature were examined, it was concluded that these studies (Neff & Faso, 2015; Riany & Ihsana, 2021; Robinson et al., 2018; Schwartzman et al., 2022; Tekinarslan & Tok, 2023) had certain limitations regarding sample sizes. Considering the worth of the subject on the psychological condition of parents and the interest in investigating these issues, it was thought that a systematic review of the relevant studies and a meta-analytic evaluation of the effect size of the emerging relationships would contribute to the literature. In addition, meta-analysis studies attract the attention of researchers and practitioners (Kürü, 2021). Therefore, another purpose of conducting this meta-analysis is to draw the attention of researchers and practitioners to this issue and to ensure that self-compassion is adapted to intervention programs to be developed, especially for parents of children with neurodevelopmental disorders. Furthermore, although there are meta-analytic studies on self-compassion and the mentioned variables (Jefferson et al., 2020; Zessin et al., 2015), it has been observed that there is no meta-analysis in the context of NDDs. Hence, the purpose of this meta-analytic review is to analyze the relationships of self-compassion of psychological distress, parenting stress, and well-being in caregivers of children with neurodevelopmental disorders and the main research question of this study is “How does self-compassion relate to psychological distress, parenting stress, and well-being in caregivers of children with certain NDDs.”

## Method

### Search Methods

This meta-analysis was conducted according to “The Preferred Reporting Items for Systematic Reviews and Meta-Analyses (PRISMA)” (Page et al., 2021a). In this phase, it was aimed to find all the studies examining the association between self-compassion and psychological distress, parenting stress, and parental well-being in the population of parents who have children with NDDs. Web of Science (WOS), Scopus, and EBSCOHost (APA PsycArticles, MEDLINE, TR Index, ERIC) electronic databases were searched in November 2023 to reach related studies. When searching the database the following keywords were used in Boolean logic: (“neurodevelopment disorder” OR “autism” OR “intellectual disability” OR “intellectually disabled” OR “learning disorder” OR “special needed” OR “ADHD” OR “attention deficit” OR “communication disorders” OR “ASD” OR “asperger” OR “developmental disabilities” OR “motor disorders” OR “Tics” OR “Tourette’s” OR “stereotypic disorders”) AND (“self-compassion” OR “self compassion” OR “selfcompassion”) AND (“psychological distress” OR “anxiety” OR “anxious” OR “distress” OR “depress*” OR “parental stress” OR “stress” OR “well-being” OR “well being”). No time limit was set on the search.

### Study Selection Process

**Fig. 1:**
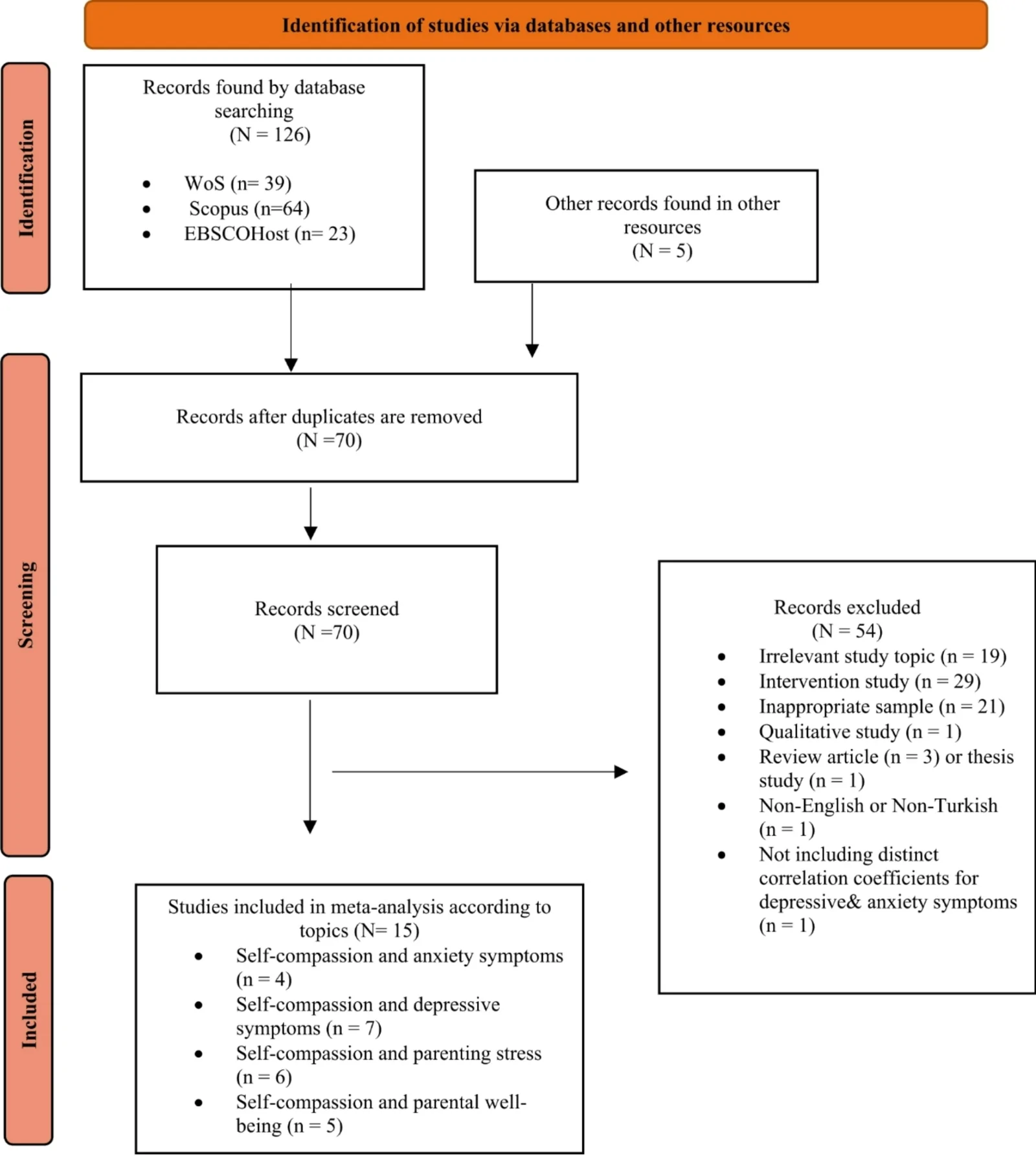
PRISMA flowchart

As a result of the research *n =* 126 studies were obtained. After the electronic database search was completed, *n =* 5 additional studies were found by searching the references of other sources. After removing the repeated studies, *n =* 70 studies remained. In order to determine which studies should be included in the meta-analysis, study titles and abstract sections were screened, considering the specified criteria. The inclusion criteria of the study were defined as follows: (i) being a quantitative study, (ii) being written in English or Turkish, (iii) being published in a peer-reviewed journal, (iv) including parents of children with NDDs as study population, (v) including correlation coefficients between self-compassion and the other variables of this meta-analysis. Exclusion criteria of the study were specified as follows: (i) being a qualitative study, (ii) being a case report, thesis, or review article, (iii) being a non-English or non-Turkish study, (iv) being an intervention study, and (v) not including appropriate data for analysis. Considering the criteria, *n =* 15 studies were evaluated as suitable for inclusion. “The PRISMA flow diagram” is given in Fig. 1.

### Study Coding Process

During the coding process, author(s), year, title, country, study variables, measurement tools, NDDs type, sample size, mean age of children and parents, and correlation coefficients were recorded in the coding table. At the end of the coding process, it was seen that there are *n =* 4 studies about self-compassion and anxiety symptoms, *n =* 7 studies about self-compassion and depressive symptoms, *n =* 6 studies about self-compassion and parenting stress, and *n =* 5 studies about self-compassion and well-being. Tables 1, 2, 3 and 4 list the primary features of the included studies.

### Statistical Analyses

Version 2.3.1.0 of Jamovi open statistical software was used to perform the analyses as it was reported to have strengths over other software packages in the meta-analysis (Eser, 2022). The decision on which effect size would be used to compute the pooled effect size was made at the onset of the statistical analysis. Since the current meta-analysis aims to reveal the relationships between variables, correlation effect size was chosen. Relatedly, Fisher’s r to z transformed correlation coefficient was used as an outcome measure in the analysis as it helps estimate the correct effect size (Kürü, 2021). The pooled effect sizes were obtained using a random effects model, which was recommended when heterogeneity was expected (Hedges & Vevea, 1998). In addition, Field and Gillett’s (2010) recommendation to use the random effects model due to the inherent nature of social sciences, regardless of the heterogeneity test result, was also taken into account. The degree of heterogeneity was ascertained using a restricted maximum likelihood estimator (i.e., tau²) (Hedges & Vevea, 1998). In addition, Cochran’s Q-test and the I² statistic are reported for heterogeneity (Cooper et al., 2019). Greater heterogeneity is indicated by higher values of I², where 0% denotes no heterogeneity, 50% moderate heterogeneity, and 75% extreme heterogeneity (Higgins, 2003). Cook’s distances and studentized residuals were used to assess whether studies were influential or whether there were any outliers within the model. Funnel plots were analyzed to investigate the possibility of publication bias. Begg and Mazumdar’s rank correlation and Egger’s regression test were used to validate the funnel plots (Page et al., 2021b).

## Results

### Descriptive Information

Four different meta-analyses were conducted to assess the relationship between self-compassion and psychological distress (anxiety-depressive symptoms), parenting stress, and parental well-being. In meta-analyses, correlation coefficients of *k =* 4 for anxiety symptoms, *k =* 7 for depressive symptoms, *k =* 7 for parenting stress, and *k =* 5 for parental well-being were used.

When the studies were analyzed in terms of the use of measurement tools, it was seen that the “Self-Compassion Scale” (*n =* 8) ( Neff, 2003b) and “Self-Compassion Scale-Short Form” (*n =* 6) (Raes et al., 2011) were widely used for measuring self-compassion. “Depression, Anxiety, Stress Scales-21- (DASS-21)” (*n =* 3) (Lovibond & Lovibond, 1995) and “Generalised Anxiety Disorder Scale (GAD-7)” (*n =* 1) (Spitzer et al., 2006) were used for measuring the anxiety symptoms of parents. For depressive symptoms of parents, “Depression, Anxiety, Stress Scales-21- (DASS-21)” (*n =* 4) (Lovibond & Lovibond, 1995), “The Patient Health Questionnaire (PHQ-9)” (*n =* 1) (Kroenke et al., 2001), and the “10-item Center for Epidemiologic Studies Short Depression Scale (CES-D)” (*n =* 2) (Andresen et al., 1994) were used. For parenting stress, “Parenting Stress Index, Fourth Edition, Short Form (PSI-4-SF)” (*n =* 3) (Abidin, 1990b), “Parental Stress Scale (PSS)” (*n =* 1) (Berry & Jones, 1995), and “Autism Parenting Stress Index (APSI)” (*n =* 1) (Silva & Schalock, 2012) were used. Lastly, parental well-being was measured by the “Subjective-Well Being Scale (SWS)” (*n =* 2), the “Psychological Well-Being Scale (PWS)” (*n =* 1), “The Satisfaction with Life Scale (SWLS)” (*n =* 1), and “Personal Well-Being Index (PWI-A)” (*n =* 1).

Regarding the sample characteristics, parents who have children with autism spectrum disorder (*n =* 10), ADHD (*n =* 1), intellectual disability and developmental disorders (*n =* 2), and intellectual disability (*n =* 2) constituted the sample of included research. Considering these numbers, it can be said that researchers have more tendency to conduct studies with the parents whose children have ASD. The study’s overall sample size was *N* = 1841. All of the studies were conducted between 2014 and 2023, which may indicate a trend in conducting studies on self-compassion and its consequences with this population group.

### The Association of Self-Compassion with Parental Anxiety

Using random effects analysis, the weighted correlation (Fisher’s r transformation) between parental self-compassion and anxiety symptoms was calculated to explore the relationship between the two variables. An overall effect size of *r* = -0.49 (*N* = 785, 95% CI: -0.66 to -0.33) was obtained based on *k =* 4 effect sizes. This overall effect size shows a moderate, negative, and significant relationship between self-compassion and parents’ anxiety symptoms, which means increased levels of self-compassion are linked to reduced anxiety symptoms. Related forest plots (Fig. 2) can be found in supplementary materials.

The heterogeneity test showed significant heterogeneity among effect sizes (Q (3) = 11.18, *p* < 0.05), and I² = 78% pointed out a high variability among studies. Studentised residuals have shown that there is no evidence of outliers. Cook’s distances revealed that none of the research was described as overly influential. To determine whether there was any publication bias, the funnel plot (Fig. 3 in supplementary materials) was analyzed, and it was observed that the funnel plot showed a roughly symmetrical shape. Also, when publication bias analyses were conducted, the rank correlation and Egger’s regression test did not reveal any funnel plot asymmetry (*p* > 0.05). Publication bias assessment values are given in Table 5 in supplementary materials.

### The Association of Self-Compassion with Parental Depression

Using random effects analysis, the weighted correlation (Fisher’s r transformation) between depressive symptoms and parental self-compassion was calculated to explore the relationship between the two variables. An overall effect size of *r* = -0.59 (*N* = 992, 95% CI: -0.68 to -0.49) was obtained based on *k =* 7 effect sizes. This effect size shows a large, negative, and significant relationship between parents’ depressive symptoms and self-compassion. That is, increased degrees of self-compassion are associated with reduced depressive symptoms. Related forest plots (Fig. 4) can be found in supplementary materials. For heterogeneity, it was seen that the Q- test is not significant (Q (6) = 11.25, *p* > 0.05), and I² = 43% pointed out a low variability among studies. Studentised residuals have shown that there is no evidence of outliers. Cook’s distances revealed that none of the research was described as overly influential. The funnel plot (Fig. 5 in supplementary materials) was analyzed to determine whether there was any publication bias, and it was observed that the funnel plot showed a roughly symmetrical shape. Also, when publication bias analyses were conducted, the rank correlation and Egger’s regression test did not reveal any funnel plot asymmetry (*p* > 0.05). For the test results, see Table 6 in the supplementary materials.

### The Association of Self-Compassion with Parenting Stress

Using random effects analysis, the weighted correlation (Fisher’s r transformation) between parenting stress and parental self-compassion was calculated to explore the association between the two variables.

An overall effect size of *r* = -0.74 (*N* = 722, 95% CI: -1.10 to -0.38) was obtained based on *k =* 7 effect sizes. For heterogeneity, the significance of the Q-test was observed (Q (6) = 208.93, *p* < 0.001), and I² = 95% pointed out a very high variability among studies. One study Bakır and Demirli (2020) showed a value larger than ± 2.69 when the studentized residuals were examined; this study could be an outlier in the context of this model. Also, Bakır and Demirli (2020)’s study can be regarded as overly influential based on Cook’s distances. Since it was determined that the study of Bakır and Demirli (2020) was overly influential and made the most significant contribution to heterogeneity, the meta-analysis was conducted a second time after this study was removed. The related forest plot, including Bakır and Demirli(2020)’s study, is given in Fig. 6 in supplementary materials.

After the meta-analysis was performed a second time, the overall effect size of *r* = -0.53 (*N* = 542, 95% CI: -0.66 to -0.41) was obtained based on *k =* 6 effect sizes. Accordingly, parental stress level and self-compassion showed a large, negative, and statistically significant association when considering the pooled effect size. That is, parents who have higher levels of self-compassion also report lower levels of parenting stress. Figure 7 shows the related forest plot in supplementary materials.

For heterogeneity, the Q-test was found to be not significant (Q (5) = 7.74, *p* > 0.05), and I² = 38% pointed out a low variability among studies. Upon analyzing the studentized residuals, it was found that all the studies had values less than or equal to ± 2.64, indicating that there was no evidence of outliers. Cook’s distances revealed that none of the research was described as overly influential.

The funnel plot (Fig. 8 in supplementary materials) was analyzed to determine whether there was any publication bias, and it was observed that the funnel plot showed a roughly symmetrical shape, indicating no publication bias. Furthermore, the funnel plot is confirmed by the Egger regression test and rank correlation (Table 7), demonstrating no asymmetry in the funnel plot (*p* > 0.05).

### The Association of Self-Compassion and Parental Well-Being

Random effects analysis was used to calculate the weighted correlation (Fisher’s r transformation) between caregivers’ self-compassion and well-being to investigate the relationship between the two variables. An overall effect size of *r* = 0.63 (*N* = 645, 95% CI: 0.46 to 0.80) was obtained based on *k =* 5 effect sizes. There is a large, positive, and significant correlation between parents’ levels of well-being and self-compassion, as seen by this pooled effect size. In other words, parents who exhibit higher degrees of self-compassion also tend to exhibit higher levels of well-being. Related forest plots (Fig. 9) can be found in supplementary materials.

The heterogeneity test showed significant heterogeneity among effect sizes (Q (4) = 14.72, *p* < 0.01), and I² = 76% pointed out a high variability among studies. Studentised residuals have shown that there is no evidence of outliers. Cook’s distances revealed that none of the research was described as overly influential.

When the funnel plot (see Fig. 10 in supplementary materials) is analyzed, it shows a roughly symmetrical shape, i.e., no publication bias. Furthermore, the Egger regression test and rank correlation confirm the funnel plot, demonstrating that there is no asymmetry in it (*p* > 0.05) (Table 8 in supplementary materials).

Finally, although publication bias was not detected in meta-analyses, the results should be interpreted carefully because of the small number of included articles (Cooper et al., 2019).

## Discussion

In the past few years, research evaluating the relationship between self-compassion and various variables such as psychological distress, parental well-being, and parenting stress has been increasing. In addition, the issue of having a child with NDDs and its consequences on parents has attracted the attention of researchers and clinicians. Although this meta-analysis aimed to examine the relevant variables across all neurodevelopmental disorders (NDDs), it was observed that studies investigating the relationship between self-compassion and psychological distress, parenting stress, and parental well-being predominantly focused on parents of children with autism spectrum disorder (ASD), followed by those with intellectual disability (ID). Additionally, due to the absence of studies in the literature examining the relationship between self-compassion and the other variables of this study within the context of communication and motor disorders, literature on these diagnostic groups could not be included in either the introduction or discussion sections. In this regard, it is important to highlight this limitation at the beginning of the discussion section. To ensure accuracy in interpretation, the findings should be considered primarily within the context of parents of children with ASD and ID, and generalizations to all NDDs should be avoided.

Within this scope, the results of the current meta-analysis demonstrated a moderately negative association between parental anxiety levels and self-compassion, suggesting that parents who practice self-compassion are less likely to exhibit symptoms of anxiety. It can be seen that the overall effect size of the relationship between parental anxiety levels and self-compassion in this meta-analysis is consistent with the results of a previous review carried out by Jefferson et al. (2020). Our results also align with the research conducted by Beer et al. (2013), which demonstrated a significant and negative correlation between self-compassion and parental anxiety levels in parents of children diagnosed with ASD. In a more recent study, Avdiu and Hyseni Duraku (2024) also found that higher self-compassion in parents of children with NDDs was associated with lower levels of anxiety and improved adaptive functioning, including better emotion regulation skills. Beer et al. (2013) stated that parents may experience significant emotional distress due to anxiety even if their anxiety levels are not at the clinical level, and this should be taken into consideration by clinicians. Having a child with a neurodevelopmental disorder can bring additional burdens to parents in terms of caregiving, meeting special education needs, taking part in the therapy process, and being exposed to stigmatization (Bessette Gorlin et al., 2016; Mitter et al., 2019). All of these NDDs- related experiences may contribute to increasing the parents’ anxiety symptoms (Pyszkowska et al., 2021). Neff and Faso (2015) states that while parents of children with ASD often experience concerns about the future and feelings of hopelessness, self-compassion seems to foster inner strength and self-assurance, enabling them to maintain a more positive perspective. Therefore, it would be meaningful to encourage parents to practice self-compassion, considering that their anxiety levels may be high during the process. However, in semi-structured interviews with adult participants in Pauley and McPherson’s (2010) study, although participants thought that self-compassion could be beneficial for depression/anxiety symptoms, some participants stated that these depression/anxiety symptoms made it difficult for them to practice self-compassion in their daily lives. Based on this, it is important to consider this challenge in self-compassion programs for parents of children with neurodevelopmental disorders.

Related to psychological distress, another finding of this meta-analysis was the large, negative, and significant relationship between parental depressive symptoms and self-compassion in parents of children with ASD and ID. This finding suggests that self-compassion helps parents to show fewer depressive symptoms. Consistent with this result, MacBeth and Gumley (2012) reported in their meta-analytic review that they found a large, negative, and significant association between being self-compassionate and experiencing depressive symptoms in the adult population. In addition, Cheung et al. (2022) stated that increased parental self-compassion was associated with decreased parental depressive symptoms. It is possible to conclude that our finding regarding the connection between parental depressive symptoms and self-compassion is consistent with the literature. Parents of children with NDDs, particularly those with ASD and ID, may exhibit depressive symptoms due to the challenges and grief they experience. However, self-compassion can enable them to recognize their negative thoughts more effectively and reduce their tendency to ruminate. They can be more accepting of their lives and their children’s situations, which may serve to reduce depressive symptoms (Neff & Faso, 2015).

Another variable related to self-compassion that was investigated was parenting stress. It was found that there is a large, negative, and significant relationship between self-compassion and parenting stress levels. This result suggests that a higher degree of self-compassion is related to a lower degree of parenting stress. Supporting our finding, Moreira et al. (2015) reported that self-compassion is negatively associated with parenting stress in their study. A more recent study by Chorão et al. (2022) found a strong, negative, and significant relationship between self-compassion and parenting stress. Therefore, the findings of the current meta-analysis are broadly consistent with previous studies in the literature. According to Bohadana et al. (2019), parents’ self-efficacy in parenting could be negatively impacted if they experience higher levels of isolation, self-criticism, and over-identification—all of which are negative aspects of self-compassion. Therefore, this may lead to a higher level of parenting stress in the caregiving relationship with a child with ASD. Conversely, when a child displays problematic behaviors or tantrums, a parent’s self-compassion helps them control their own emotions, which makes the scenario seem less upsetting and often shields the parent from going through a stressful parenting period (Neff & Faso, 2015).

Nevertheless, Constantakes (2021) revealed that among parents of children with ASD, there was no apparent correlation between self-compassion and parenting stress. However, the researcher also states that the time of the study may be a possible confounding variable since the study was conducted during the COVID-19 pandemic. During COVID-19, parenting stress may have decreased in importance in favor of stress connected to child education and infection prevention. Despite this contradictory finding, it is believed that self-compassion positively contributes to a decrease in parenting stress.

Parental well-being was the last variable evaluated about self-compassion. Analysis has shown a large, significant, and positive correlation between parental well-being and self-compassion. In other words, parents of children with ASD/ID who exhibit higher degrees of self-compassion also tend to show higher levels of well-being—in support of this finding, Zessin et al. (2015) presented a moderate and positive relationship between self-compassion and well-being in their meta-analysis study. More relevant to our study, Ahmed and Raj (2023) conducted a self-compassion-based online intervention for parents of children with developmental disabilities during the COVID-19 pandemic and reported that parents’ well-being levels increased significantly after the intervention. According to research, self-compassion can improve well-being by encouraging self-kindness, reducing self-judgment, and improving emotional resilience. When taken together, these factors support the overall well-being of parents of children with ASD by helping them feel less stressed and depressed (Ahmed & Raj, 2023).

Both clinical observations reflected in the literature and studies have shown that having a child with NDDs creates an unexpected career for parents in terms of being involved in the child’s therapy process, managing the child’s problematic behaviors, and supporting the progress of the abilities at the same time (Rafferty et al., 2020). Because of this unexpected career and the challenging nature of these disorders, parents can often exhibit feelings of hopelessness, fatigue, anxiety, grief, and self-judgment. Furthermore, they frequently expressed worries about their children’s circumstances in the future, including whether or not their children will develop as well as their peers, whether or not they will be able to live independently, and what will happen to them when the caregivers pass away (Ahmed & Raj, 2023; DePape & Lindsay, 2015; Lashewicz et al., 2019; Neff & Faso, 2015; Shorey & Pereira, 2023). These worries could lead to an increased tendency to over-identify with the concerns as it projects the negative aspects of self-compassion. In light of all these reasons and the findings mentioned above, it is seen that self-compassion is a critical variable that must be considered in studies conducted with these families. The concept of self-compassion must also be emphasized in intervention programs designed to improve parental well-being in parents of children with neurodevelopmental disorders by lowering anxiety-depression levels and parenting stress. Given the critical role of self-compassion in mitigating psychological distress and enhancing well-being, its integration into research and intervention programs becomes increasingly essential. This study provides a valuable contribution as it appears to be one of the first meta-analyses to explore the relationship between self-compassion and anxiety, depression, parenting stress, and parental well-being especially in parents of children with ASD and ID. Moreover, the study offers a more holistic view by examining both adverse psychological outcomes (depression, anxiety, and parenting stress) and positive indicators of psychological health (well-being). Moreover, the findings suggest that therapies focused on self-compassion could enhance the psychological well-being of these parents while potentially reducing their psychological distress and parenting stress.

Finally, it is essential to mention some limitations of this current study and suggestions for further studies. The most significant limitation of this meta-analytic evaluation can be attributed to the comparatively small number of studies included in the meta-analysis. It is seen that the number of effect sizes included in meta-analyses varies between *k =* 4 and *k =* 7. The moderator analyses were not performed because of the small number of effect sizes (Boemo et al., 2022; Jefferson et al., 2020). Although the introduction section presents findings about the correlational nature between self-compassion and psychological distress, parenting stress, and well-being, few studies have examined potential mediating or moderating variables in the relationship between these variables (Emmanuel et al., 2022; Maridal et al., 2021; Nagase et al., 2024; Nemati et al., 2023; Schiltz et al., 2022; Torbet et al., 2019). Therefore, these studies could only be mentioned to a limited extent. At this point, further research is needed to explore potential mediating and moderating variables in the relationship between self-compassion and other variables of this meta-analysis. Considering this limitation, readers are encouraged to interpret our findings within this context. In addition, it is believed that for providing clinical interventions for parents, it is crucial to take into account and evaluate the factors that might act as moderators in the association between parental anxiety-depression symptoms, parenting stress, well-being, and self-compassion. Another limitation was the widely used measurement method of self-compassion. In the studies included in this meta-analysis, self-compassion was mainly assessed using the “Self-Compassion Scale” (SCS) developed by Neff (2003b). It is seen that the correlation effect sizes in the studies included in the current meta-analysis belong to the scale’s total score. MacBeth and Gumley (2012) state that the correlation coefficients calculated based on the total score are insufficient to understand whether the relationships revealed are due to high scores obtained from the positive dimension of self-compassion (e.g., high self-kindness score) or low scores obtained from the negative dimension (e.g., low over-identification). Therefore, this meta-analysis also shares this mentioned limitation. This limitation will likely be overcome when more quantitative research on self-compassion is conducted by independently meta-analyzing the correlation coefficients derived from the scale’s positive and negative aspects. The other significant limitation of this meta-analysis is the limited representation of all neurodevelopmental disorders. Among the included studies, ten focused on parents of children with ASD, four on parents of children with ID, and only one on parents of children with ADHD. There are no studies involving parents of children with specific learning disorders, communication disorders, and motor disorders. This imbalance restricts the generalizability of the findings to all parents of children with neurodevelopmental disorders. Considering the findings that the experiences of parents of children with autism spectrum disorder may not fully overlap with the experiences of parents of children with other neurodevelopmental disorders (Dovgan & Mazurek, 2018; Emmanuel et al., 2022), one should be cautious in generalizing the results of this study to all neurodevelopmental disorders. However, given that self-compassion was associated with positive psychological outcomes in a parent group characterized by elevated parenting stress and psychological distress (Craig et al., 2016; Maridal et al., 2021; Nagase et al., 2024; Neff & Faso, 2015; Pyszkowska et al., 2021), it is reasonable to suggest that self-compassion may yield similar benefits for parents of children with other neurodevelopmental disorders. To substantiate this interpretation, further research is needed, specifically including parents of children with other neurodevelopmental disorder subtypes.

Finally, parents’ self-compassion levels were obtained using scales in quantitative studies. However, conducting qualitative studies to obtain more detailed information about parents’ self-compassion experiences may provide a better understanding of these experiences and provide more comprehensive guidance on encouraging self-compassion practices in parents.

**Table 1:** Study characteristics of included studies about the relationship between self-compassion and parents’ anxiety symptoms

Author	Country	NDD Type	*N*	Age of Parents	Age of Children	*r*	Measurement	
Chan et al. (2020a, b	China	ASD	121	M_age_ = 34.70 (Mothers) M_age_ = 36.98 (Fathers)	M_age_ = 5.94	-0.37^**^	SCS-SF; DASS-21	
Chan et al. (2022)	China	ASD	381	M_age_ = 45.78	M_age_ = 11.61	-0.50^***^	SCS-SF; GAD-7	
Pyszkowska et al. (2021)	Poland	ASD	233	M_age_ = 39.18	M_age_ = 10.15	-0.59^***^	SCS-SF; DASS-21	
Schwartzman et al. (2022)	USA	ASD	50	M_age_ = 40.6	M_age_ = 6.9	-0.24	SCS-SF; DASS-21	

Note. M_age_ = Mean age, N = Sample size, r = Correlation coefficient, SCS-SF = Self-Compassion Scale- Short Form (Raes et al., 2011), DASS-21 = Depression, Anxiety, Stress Scales-21 (Lovibond & Lovibond, 1995), GAD-7 = Generalised Anxiety Disorder Scale (Spitzer et al., 2006), ^*^p < 0.05; ^**^p < 0.01; ^***^p < 0.001

**Table 2:** Study characteristics of included studies about the relationship betweenself-compassion and parents’ depressive symptoms

Author	Country	NDD Type	*N*	Age of Parents	Age of Children	*r*		Measurement
Chan et al. (2020a, b	China	ASD	121	M_age_ = 34.70 (Mothers) M_age_ = 36.98 (Fathers)	M_age_ = 5.94	-0.53^**^		SCS-SF; DASS-21
Chan et al. (2022)	China	ASD	381	M_age_ = 45.78	M_age_ = 11.61	-0.48^***^		SCS-SF; PHQ-9
Pyszkowska et al. (2021)	Poland	ASD	233	M_age_ = 39.18	M_age_ = 10.15	-0.54^***^		SCS-SF; DASS-21
Schwartzman et al. (2022)	USA	ASD	50	M_age_ = 40.6	M_age_ = 6.9	-0.43^**^		SCS-SF; DASS-21
Ivins-Lukse and Lee (2021)	USA	Intellectual disability and developmental disorder (IDD)	100	M_age_ = 46.97	M_age_ = 17.22	-0.66^***^		SCS; CES-D-10
Neff and Faso (2015)	USA	ASD	51	M_age_ = 39.90 (Mothers) M_age_ = 42.27 (Fathers)	Range = 4–12	-0.65^**^		SCS; CES-D-10
Robinson et al. (2018)	USA	IDD	56	M_age_ = 56.5	M_age_ = 23	-0.33^*^		SCS-SF; DASS-21

Note. M_age_ = Mean age, N = Sample size, r = Correlation coefficient, SCS = Self-Compassion Scale ( Neff, 2003b), SCS-SF = Self-Compassion Scale- Short Form (Raes et al., 2011), DASS-21 = Depression, Anxiety, Stress Scales-21 (Lovibond & Lovibond, 1995), PHQ-9 = The Patient Health Questionnaire (Kroenke et al., 2001), CES-D-10 = The 10-item Center for Epidemiologic Studies Short Depression Scale (Andresen et al., 1994), ^*^p < 0.05; ^**^p < 0.01; ^***^p < 0.001

**Table 3:** Study characteristics of included studies about the relationship between self-compassion and parenting stress

Author	Country	NDD Type	*N*	Age of Parents	Age of Children	*r*	Measurement
Bakır and Demirli (2020)	Turkey	Learning disorder (LD), ASD, ID	180	No information	No information	-0.94^***^	SCS; PSI-SF
Bohadana et al. (2019)	Australia	ASD	139	M_age_ = 39.49 (Mothers) M_age_ = 35.16 (Fathers)	M_age_ = 8.94	-0.41^**^	SCS; PSI-SF
Neff and Faso (2015)	USA	ASD	51	M_age_ = 39.90 (Mothers) M_age_ = 42.27 (Fathers)	Range = 4–12	-0.66^**^	SCS PSI-SF
Schwartzman et al. (2022)	USA	ASD	50	M_age_ = 40.6	M_age_ = 6.9	-0.58^***^	SCS-SF; PSI-SF
Riany and Ihsana (2021)	Indonesia	ASD and ADHD	*N* = 34 (ASD Mothers) *N* = 31 (ADHD Mothers)	M_age_ = 37.65	M_age_ = 9.12	-0.38 (ASD)^*^ -0.59 (ADHD)^**^	SCS; PSS
Torbet et al. (2019)	Australia	ASD	237	M_age_ = 39.97	M_age_ = 8.73	-0.42^**^	SCS; APSI

Note. M_age_ = Mean age, N = Sample size, r = Correlation coefficient, SCS = Self-Compassion Scale ( Neff, 2003b), SCS-SF = Self-Compassion Scale- Short Form (Raes et al., 2011), PSS = Parental Stress Scale (Berry & Jones, 1995), APSI = Autism Parenting Stress Index (Silva & Schalock, 2012), PSI-4-SF = Parenting Stress Index, Fourth Edition, Short Form (Abidin, 1990b), ^*^p < 0.05; ^**^p < 0.01; ^***^p < 0.001

**Table 4:** Study characteristics of included studies about the relationship between self-compassion and parental well-being

Author	Country	NDD Type	*N*	Age of Parents	Age of Children	*r*	Measurement
Duran and Barlas (2014)	Turkey	ID	101	M_age_ = 38.8	No information	0.65^**^	SCS; SWS
Nemati et al. (2023).	Azerbaijan	ASD	101	M_age_ = 27.3	No information	0.42^**^	SCS; PWS
Tati (2017)	Indonesia	ID	136	No information	No information	0.42^***^	SCS; SWLS
Torbet et al. (2019)	Australia	ASD	237	M_age_ = 39.97	M_age_ = 8.73	0.55^**^	SCS PWI-A
Tekinarslan and Tok (2023)	Turkey	ID and ASD	70	M_age_ = 36	No information	0.72^***^	SCS-SF; SWS

Note. M_age_ = Mean age, N = Sample size, r = Correlation coefficient, ID = Intellectual Disability, SCS = Self-Compassion Scale ( Neff, 2003b), SCS-SF = Self-Compassion Scale- Short Form (Raes et al., 2011), PWI-A = Personal Well-Being Index (Cummins et al., 2003), PWS = Psychological Well-Being Scale (Ryff & Keyes, 1995), SWLS = The Satisfaction with Life Scale (Diener et al., 1985), SWS = Subjective-Well Being Scale (Dost, 2005), ^*^p < 0.05; ^**^p < 0.01; ^***^p < 0.001

## Electronic supplementary material

Below is the link to the electronic supplementary material.
