# Supplementary material for: Self-Compassion and Psychological Health of Parents: A Meta-Analysis Focused on Some Neurodevelopmental Disorders
Source: J Autism Dev Disord. 2025 Apr 21;56(10):3828–43. doi: 10.1007/s10803-025-06841-9 (PMC13538213; doi:10.1007/s10803-025-06841-9)
Supplement: Supplementary file 1 [file 10803_2025_6841_MOESM1_ESM.docx]

**Table 5** Publication Bias Assessment Values for Self-Compassion and Anxiety Symptoms Meta-Analysis

|  | value | *p* |
| --- | --- | --- |
| Begg and Mazumdar Rank Correlation | 0.667 | > .05 |
| Egger’s Regression | 1.949 | > .05 |

**Table 6** Publication Bias Assessment Values for Self-Compassion and Depressive Symptoms Meta-Analysis

|  | value | *p* |
| --- | --- | --- |
| Begg and Mazumdar Rank Correlation | -0.048 | > .05 |
| Egger’s Regression | 0.037 | > .05 |

**Table 7** Publication Bias Assessment Values for Self-Compassion and Parenting Stress

|  | value | *p* |
| --- | --- | --- |
| Begg and Mazumdar Rank Correlation | -0.333 | > .05 |
| Egger’s Regression | -1.797 | > .05 |

**Table 8** Publication Bias Assessment Values for Self-Compassion and Well-Being

|  | value | *p* |
| --- | --- | --- |
| Begg & Mazumdar Rank Correlation | 0.527 | > .05 |
| Egger’s Regression | 1.002 | > .05 |

**Fig. 2** Forest Plot Towards Examination of Relationship Between Self-Compassion and Parents’ Anxiety Symptoms

**Fig. 3** Funnel Plot Analysis of Self-Compassion and Anxiety Symptoms Meta-Analysis

**Fig. 4** Forest Plot Towards Examination of Relationship Between Self-Compassion and Parents’ Depressive Symptoms

**Fig. 5** Funnel Plot Analysis of Self-Compassion and Depressive Symptoms Meta-Analysis

**Fig. 6** Forest Plot Analysis of Self-Compassion and Parenting Stress Symptoms

**Fig. 7** Forest Plot Towards Examination of Relationship Between Self-Compassion and Parenting Stress

**Fig. 8** Funnel Plot Analysis of Self-Compassion and Parenting Stress

**Fig. 9** Forest Plot Towards Examination of Relationship Between Self-Compassion and Parental Well-Being

**Fig. 10** Funnel Plot Analysis of Self-Compassion and Parental Well-Being
